# Alpine Meadow Fractional Vegetation Cover Estimation Using UAV-Aided Sentinel-2 Imagery

**DOI:** 10.3390/s25144506

**Published:** 2025-07-20

**Authors:** Kai Du, Yi Shao, Naixin Yao, Hongyan Yu, Shaozhong Ma, Xufeng Mao, Litao Wang, Jianjun Wang

**Affiliations:** 1 Qinghai Provincial Key Laboratory of Physical Geography and Environmental Process, College of Geographical Science, Qinghai Normal University, Xining 810008, China; 20211028@qhnu.edu.cn (K.D.); 20224711338@stu.qhnu.edu.cn (Y.S.); maoxufeng@yeah.net (X.M.); 2 Key Laboratory of Tibetan Plateau Land Surface Processes and Ecological Conservation (Ministry of Education), Qinghai Normal University, Xining 810008, China; 3 Southern Qilian Mountain Forest Ecosystem Observation and Research Station of Qinghai Province, Huzhu 810500, China; 4 Qinghai Forestry Engineering Supervision Center Co., Ltd., Xining 810008, China; yao810418@163.com; 5 Service and Support Center of Qilian Mountain National Park in Qinghai, Xining 810008, China; qhyuhy@163.com; 6 Yeniugou Forest Farm, Qilian 810499, China; qlxnyglc_3471@163.com; 7 The College of Forestry, Beijing Forestry University, Beijing 100083, China; ltwang2018@163.com; 8 Research Institute of Forestry Policy and Information, Chinese Academy of Forestry, Beijing 100091, China

**Keywords:** Qinghai–Tibet Plateau, fractional vegetation cover, machine learning, pixel dichotomy model, shapley additive explanations

## Abstract

Fractional Vegetation Cover (FVC) is a crucial indicator describing vegetation conditions and provides essential data for ecosystem health assessments. However, due to the low and sparse vegetation in alpine meadows, it is challenging to obtain pure vegetation pixels from Sentinel-2 imagery, resulting in errors in the FVC estimation using traditional pixel dichotomy models. This study integrated Sentinel-2 imagery with unmanned aerial vehicle (UAV) data and utilized the pixel dichotomy model together with four machine learning algorithms, namely Random Forest (RF), Extreme Gradient Boosting (XGBoost), Light Gradient Boosting Machine (LightGBM), and Deep Neural Network (DNN), to estimate FVC in an alpine meadow region. First, FVC was preliminarily estimated using the pixel dichotomy model combined with nine vegetation indices applied to Sentinel-2 imagery. The performance of these estimates was evaluated against reference FVC values derived from centimeter-level UAV data. Subsequently, four machine learning models were employed for an accurate FVC inversion, using the estimated FVC values and UAV-derived reference FVC as inputs, following feature importance ranking and model parameter optimization. The results showed that: (1) Machine learning algorithms based on Sentinel-2 and UAV imagery effectively improved the accuracy of FVC estimation in alpine meadows. The DNN-based FVC estimation performed best, with a coefficient of determination of 0.82 and a root mean square error (RMSE) of 0.09. (2) In vegetation coverage estimation based on the pixel dichotomy model, different vegetation indices demonstrated varying performances across areas with different FVC levels. The GNDVI-based FVC achieved a higher accuracy (RMSE = 0.08) in high-vegetation coverage areas (FVC > 0.7), while the NIRv-based FVC and the SR-based FVC performed better (RMSE = 0.10) in low-vegetation coverage areas (FVC < 0.4). The method provided in this study can significantly enhance FVC estimation accuracy with limited fieldwork, contributing to alpine meadow monitoring on the Qinghai–Tibet Plateau.

## 1. Introduction

Alpine meadows, as the primary ecosystem of the Qinghai–Tibet Plateau, cover more than 35% of the plateau area and play a crucial role in ecological security and livestock production [1]. Vegetation conditions in alpine meadows significantly impact carbon storage, soil and water conservation [2], and biodiversity conservation [3]. In recent years, with climate change and increased grazing intensity [4], the precise monitoring of alpine meadow vegetation has become critical for ecological protection. Fractional vegetation cover (FVC) reflects the horizontal density of vegetation and serves as an essential basis for grassland carbon storage estimations and health assessments [5].

FVC retrieval methods can be divided into two categories: methods based on the pixel dichotomy model [6] and machine learning [7]. As a classical method for FVC inversion, the pixel dichotomy model is based on the assumption that a mixed pixel can be linearly decomposed into pure vegetation and pure soil components and calculates FVC through endmember values (such as NDVI_veg_ and NDVI_soil_) [8]. The model was employed to estimate the sparse desert vegetation coverage in the arid area of southern Xinjiang and compare the inversion results with the measured vegetation coverage in the field. It was found that the pixel binary model could derive high-accuracy results but required pure endmembers [9]. Yan et al. evaluated the performance of FVC estimation by combining the pixel dichotomy model with various vegetation indices (VI) using radiative transfer simulations. They pointed out that the inversion accuracy of different vegetation indices varied depending on vegetation conditions [10]. Machine learning-based methods focus on establishing relationships between remote sensing features and ground-measured vegetation coverage. These approaches generally achieve higher inversion accuracy for vegetation coverage but require extensive ground surveys. Li et al. employed machine learning algorithms using data from 920 ground observation points to estimate FVC in alpine grasslands, achieving a coefficient of determination of 0.867 for the inversion results [11]. Xu et al. compared the performance of the Random Forest model and the Linear Spectral Mixture Model in estimating shrub coverage and reported that the Random Forest model produced more accurate results [12]. By constructing and comparing multicollinearity and non-multicollinearity contexts of vegetation indices, Derraz et al. demonstrated that the performance and overfitting/underfitting of machine learning models were better in multicollinearity contexts than in non-multicollinearity contexts, and that multicollinearity did not affect the model confidence [13].

Despite the widespread and successful application of both the pixel dichotomy model and machine learning algorithms for FVC estimation under various conditions, several challenges remain that may limit the estimation accuracy. For the pixel dichotomy model, the primary issue is to extract pure endmembers and handle the spectral variability of the endmembers [14], especially since exposed rocks and freeze–thaw soils in alpine meadow regions have high reflectance, which causes NDVI_soil_ to be overestimated. In machine learning models, the nonlinear relationships between feature variables and the target variable can be affected by factors such as atmospheric conditions and sun–sensor geometry, which pose a challenge to the robustness of the models [15]. To mitigate the variability of endmember spectra and the effects of illumination and atmospheric conditions, a straightforward approach is to generate new features through feature transformation to replace the original spectral reflectance [16,17]. Given the differing abilities of vegetation indices to suppress interference [18], an effective strategy involves initially estimating FVC using the pixel dichotomy model in combination with various vegetation indices. These preliminary FVC estimates are then used as feature variables in machine learning models, offering the potential to improve the accuracy of FVC inversion.

In addition, incorporating ground measurements can improve the accuracy of FVC estimation. However, in alpine meadow regions, high elevation and poor accessibility make field surveys challenging, resulting in high time and economic costs. With the development of unmanned aerial vehicle (UAV) remote sensing, it has become feasible to obtain centimeter-level imagery at low cost [19], which presents an opportunity for the accurate extraction of FVC. Riihimäki et al. employed UAV imagery and optical satellite data to estimate the multi-scale vegetation coverage in the Arctic tundra, demonstrating that UAV imagery can train satellite data and examine vegetation over large regions [20]. Chen et al. proposed that UAVs provided more accurate and efficient vegetation coverage estimation at satellite image pixel scales than traditional ground survey methods [21].

Therefore, this study first evaluated the performance of FVC estimation based on the pixel dichotomy model and various vegetation indices and subsequently, proposed a method for alpine meadow FVC inversion by integrating Sentinel-2 and UAV imagery. The specific objectives are as follows: (1) to assess the performance of the pixel dichotomy model combined with various vegetation indices for FVC estimation; (2) to evaluate the accuracy of FVC inversion using different machine learning algorithms by integrating Sentinel-2 imagery with UAV data. The rest of this paper is structured as follows: Section 2 introduces the study area and sample plots, as well as the data processing and analysis methods; in Section 3, the performance of the pixel dichotomy model and the four machine learning models for FVC estimation is evaluated; a discussion about the FVC estimation results is presented in Section 4; and the conclusions are drawn in Section 5.

## 2. Materials and Methods

### 2.1. Study Area

**Figure 1:**
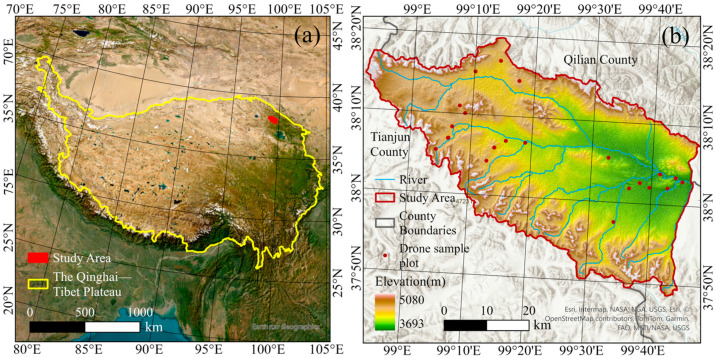
(**a**) Location of the study area in the Qinghai–Tibet Plateau; (**b**) distribution of UAV sample plots.

This study focused on the alpine meadow region located on the northeastern margin of the Qinghai–Tibet Plateau, situated in the upper reaches of the Datong River, between 98.52° E–99.45° E and 38.16° N–33.75° N. The study area covers approximately 2435 km^2^, with an average elevation exceeding 4000 m. It is characterized by a plateau frigid climate, with a mean annual temperature of −8.3 °C and an average annual precipitation of about 360 mm. The region is susceptible to climate change, with a fragile ecosystem dominated by alpine meadow vegetation. The vegetation composition is relatively simple, with limited species diversity. To comprehensively represent the FVC levels of alpine meadows in the study area, 23 UAV sample plots (50 m × 50 m) were established based on vegetation growth conditions and elevation distribution. The location of the study area and distribution of sample plots are shown in Figure 1.

### 2.2. UAV Data Collection and Processing

**Figure 2:**
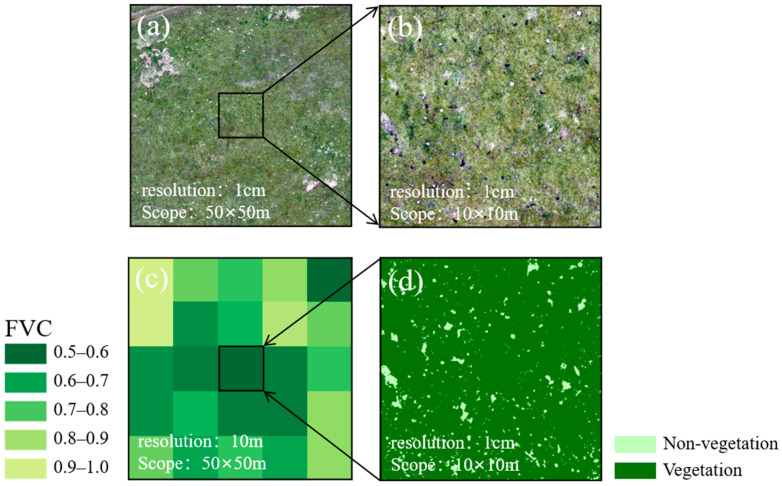
Process of FVC extraction within the Sentinel-2 pixel range. (**a**) 50 m × 50 m UAV plot orthophoto; (**b**) 10 m × 10 m UAV plot orthophoto; (**c**) FVC with a spatial resolution of 10 m; (**d**) vegetation and non-vegetation binary classification results from (**b**).

In August 2024, UAV data collection was completed for the 23 observation plots using the DJI Mavic 3 drone in this study. During data collection, the forward and side overlap rates of the flight paths were 70% and 80%, respectively. To ensure the accurate identification of vegetated and non-vegetated areas, the UAV was operated at a flight altitude of 21.7 m, yielding imagery with a spatial resolution of 1 cm [11,22]. Based on the captured UAV images and DJI Terra, orthophotos with a spatial resolution of 1 cm and a coverage area of 50 m × 50 m were generated for each plot. Subsequently, a Python 3.10-based program was used to perform a binary classification on the orthophoto, separating the vegetated and non-vegetated areas. Finally, to match the spatial resolution of Sentinel-2, the binary orthophotos were resampled to 10 m × 10 m, thereby providing vegetation coverage information corresponding to the Sentinel-2 pixels, as shown in Figure 2.

### 2.3. Sentinel-2 Data Collection and Processing

Sentinel-2 L2A surface reflectance data was utilized for FVC retrieval. Firstly, a Sentinel-2 image dataset from August 2024, corresponding to the UAV data acquisition period, was constructed on the Google Earth Engine (GEE) platform. Then, cloud-free imagery covering the entire study area was obtained through cloud cover filtering as well as image mosaicking and was subsequently used to calculate the various vegetation indices. Based on the relevant literature [10,19], nine vegetation indices were used to estimate the vegetation coverage, namely the Normalized Difference Vegetation Index (NDVI), Enhanced Vegetation Index (EVI), Red Edge Normalized Difference Vegetation Index (RENDVI), Simple Ratio (SR), Normalized Difference Water Index (NDWI), Green Normalized Difference Vegetation Index (GNDVI), Infrared Simple Ratio (ISR), Vogelmann Red Edge Index (VREI), and Near-Infrared Reflectance of Vegetation (NIRv). Since the spectral band data primarily used for FVC estimation, including red, green, blue, and near infrared, have a spatial resolution of 10 m, the data of the red edge band and the shortwave infrared band were resampled to 10 m to ensure consistency. The calculation formula and corresponding references for the various vegetation indices are shown in Table 1.

### 2.4. The FVC Estimation by Integrating Sentinel-2 and UAV Data

**Figure 3:**
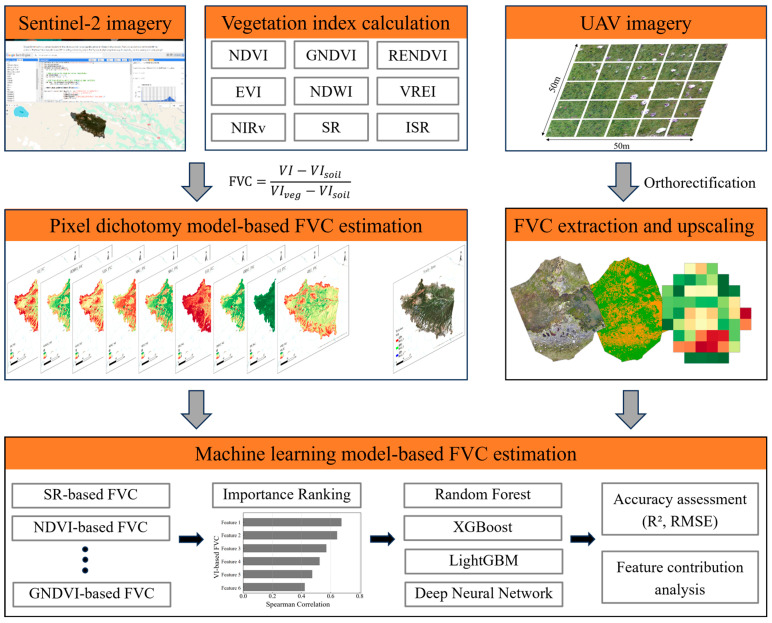
Workflow of this study.

This study proposed a method for accurately estimating FVC using high-resolution UAV imagery to assist Sentinel-2 data (Figure 3). First, the FVC was estimated using the pixel dichotomy model based on Sentinel-2 imagery and nine vegetation indices. The accuracy and applicability of the FVCs derived from the different indices were evaluated by comparison with reference FVCs extracted from centimeter-level UAV imagery using a binarization approach. Subsequently, the FVC estimates derived from different vegetation indices were used as feature variables for machine learning-based FVC estimation. Spearman correlation was utilized to rank feature importance, and features with a correlation above 0.6 were selected. Finally, four machine learning algorithms (Random Forest (RF), Extreme Gradient Boosting (XGBoost), Light Gradient Boosting Machine (LightGBM) and Deep Neural Network (DNN)) were employed to estimate the FVC accurately in the alpine meadow regions. The contribution of each feature to the estimation results was evaluated using the SHapley Additive exPlanations (SHAP).

#### 2.4.1. Pixel Dichotomy Model-Based FVC Estimation

The pixel dichotomy model assumes that a mixed pixel consists of vegetation and soil components, with its spectral information represented as a linear combination of pure vegetation and soil endmembers, weighted by their respective area proportions [32]. Therefore, the FVC of a pixel can be estimated based on the similarity between its spectral information and that of the vegetation and soil endmembers, as shown in Equation (1). The study estimated the alpine meadow FVC using nine vegetation indices (listed in Table 1) based on the pixel dichotomy model and evaluated the performance of each index.

(1) $$FVC=\frac{VI-{VI}_{soil}}{{VI}_{veg}-{VI}_{soil}}$$

where VI is the vegetation index value of the pixel; VI_veg_ is the vegetation index value of the pure vegetation pixels; and VI_soil_ is the vegetation index value of the pure bare soil pixels. This study selected 5% and 95% as the confidence intervals for determining VI_veg_ and VI_soil_ [33].

#### 2.4.2. Machine Learning-Based FVC Estimation

Four machine learning algorithms, including RF, XGBoost, LightGBM, and DNN, were employed to estimate the FVCs in this study.

RF is a widely used Bagging-based ensemble learning algorithm [34]. During model training, multiple subsets are generated from the training dataset through bootstrap sampling and used to train individual decision trees independently. In the prediction phase, the final output is obtained by averaging the predictions from all trees in the Forest.

XGBoost is a Boosting-based ensemble learning algorithm that incrementally enhances the model’s predictive performance using a sequence of decision trees [35]. Each new tree is built by minimizing the residuals of the previous iteration.

LightGBM is an efficient gradient boosting framework that employs histogram-based decision tree algorithms and a “leaf-wise” growth strategy to enhance training speed and memory efficiency significantly [36].

DNN learns complex mapping relationships between input features and target variables through multiple nonlinear transformations [37]. Its core components include fully connected layers, activation functions, and optimization algorithms.

In the machine learning models, the FVC derived from binary classification and the upscaling of UAV imagery served as the ground truth. At the same time, the FVCs estimated from Sentinel-2 imagery using various vegetation indices and the pixel dichotomy model served as the feature variables. As a preprocessing step, all feature values were normalized to ensure uniformity. To enhance model performance and efficiency, a Spearman correlation analysis was applied for feature selection, and Bayesian optimization was used to fine-tune the key parameters of the four models [38,39]. Ten-fold cross-validation was employed to evaluate the accuracy of FVC estimation in this study.

### 2.5. Shapley Additive Explanations (SHAP) Method

Due to the “black box” inversion process, machine learning models often suffer from the poor interpretability of their results, which limits their broader application. The Shapley Additive Explanation (SHAP) algorithm, as a game theory method, quantitatively explains the positive or negative contribution of each input feature to the model output by computing the Shapley value for each sample, thus enabling a detailed interpretability analysis of the model [40]. This study employed the SHAP algorithm to analyze the feature contributions in the best machine learning-based FVC estimation, aiming to assess the influence of the FVC derived from different vegetation indices on the inversion results.

### 2.6. Evaluation Metrics

The study used the coefficient of determination (R^2^, Equation (2)) and Root Mean Square Error (RMSE, Equation (3)) to assess model accuracy, quantifying the consistency between the predicted FVC and the extracted FVC based on drone imagery as well as evaluating model generalization capabilities.

(2) $$R^{2}=1-\frac{\sum_{i=1}^{n}{\left(y_{i}-\hat{y_{i}}\right)}^{2}}{\sum_{i=1}^{n}{\left(y_{i}-\bar{y_{i}}\right)}^{2}}$$

(3) $$RMSE=\sqrt{\frac{1}{n}\sum_{i=1}^{n}{\left(y_{i}-\hat{y_{i}}\right)}^{2}}$$

where $y_{i}$ and $\hat{y_{i}}$ are the extracted and predicted FVC values, respectively; $\bar{y_{i}}$ is the mean of the extracted FVC values, and n is the total number of samples.

## 3. Results

### 3.1. Pixel Dichotomy Model-Based FVC Estimation Analysis

**Figure 4:**
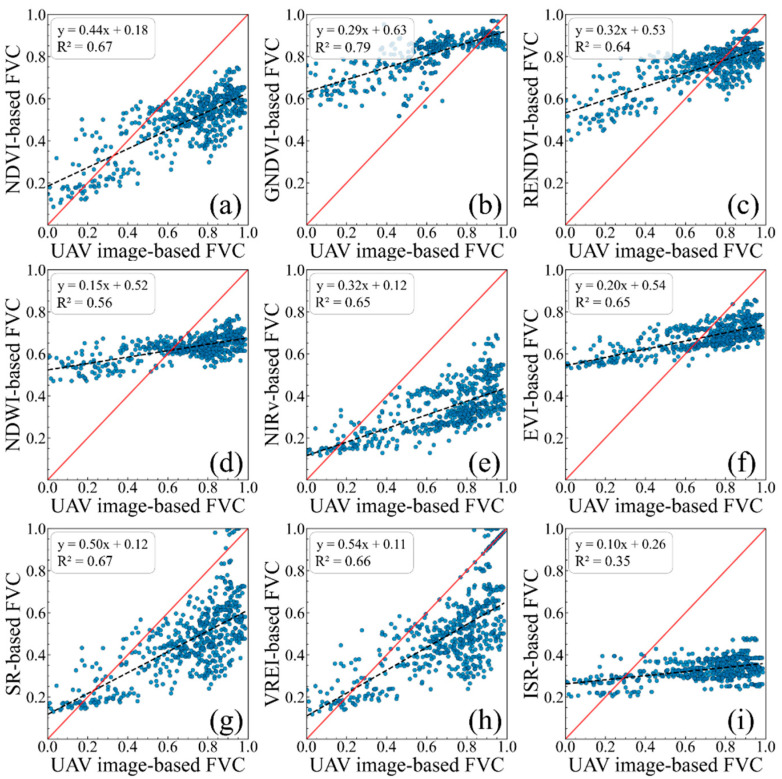
The linear regression relationship between the UAV image-based FVC and the VI-based FVC. (**a**) NDVI-based; (**b**) GNDVI-based; (**c**) RENDVI-based; (**d**) NDWI-based; (**e**) NIRv-based; (**f**) EVI-based; (**g**) SR-based; (**h**) VREI-based; (**i**) ISR-based. The red solid and the black dashed line represent the 1:1 and the regression lines, respectively.

This study individually estimated FVC by integrating the pixel dichotomy model with nine vegetation indices. The coefficients of determination and root mean square error between the estimated FVC and the UAV-derived FVC were computed to assess the performances of the various vegetation indices in estimating the alpine meadow FVC. The scatter plots illustrating the relationship between the VI-based FVC and the UAV image-based FVC are shown in Figure 4.

Figure 4 showed that the performance of the FVCs estimated using different vegetation indices varied, with the coefficient of determination between the VI-derived FVC and the UAV-derived FVC ranging from 0.35 to 0.79. Among them, except for the FVC estimates based on NDWI and ISR, which showed relatively low coefficients of determination, the other seven vegetation indices achieved R^2^ values above 0.64. Notably, the FVC estimated using GNDVI achieved the highest coefficient of determination, reaching 0.79. However, the different vegetation indices still exhibited varying degrees of overestimation or underestimation when estimating FVC. Specifically, NDVI, NIRv, SR, and VREI tend to underestimate areas with high vegetation coverage. In contrast, GNDVI, RENDVI, and EVI tend to overestimate areas with low vegetation coverage.

**Figure 5:**
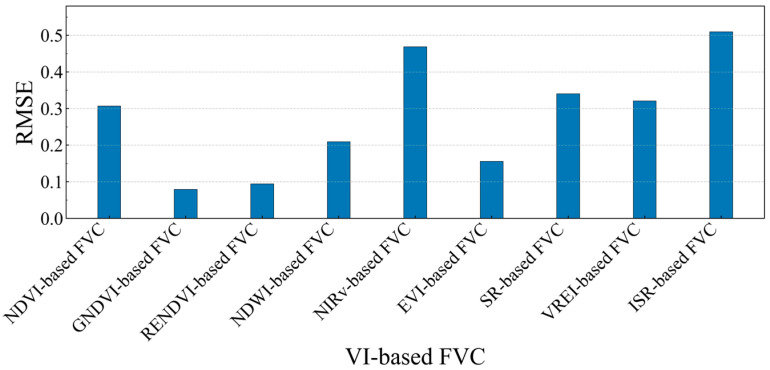
The Root Mean Square Error (RMSE) of FVC estimates based on different vegetation indices.

Figure 5 presents the root mean square error of the FVC estimates based on the different vegetation indices. The FVC estimated using GNDVI showed the lowest root mean square error at 0.08, while the estimate based on ISR had the highest root mean square error, reaching 0.51. The root mean square error of the vegetation coverage estimates for most of the vegetation indices ranged between 0.08 and 0.34. Considering both the coefficient of determination and root mean square error, the FVC estimates based on the different vegetation indices exhibited their strengths and weaknesses. Thus, using the FVCs derived from multiple indices as predictor variables in machine learning models has the potential to improve the accuracy of vegetation coverage estimation.

### 3.2. Machine Learning Model-Based FVC Estimation Analysis

#### 3.2.1. Feature Selection

**Figure 6:**
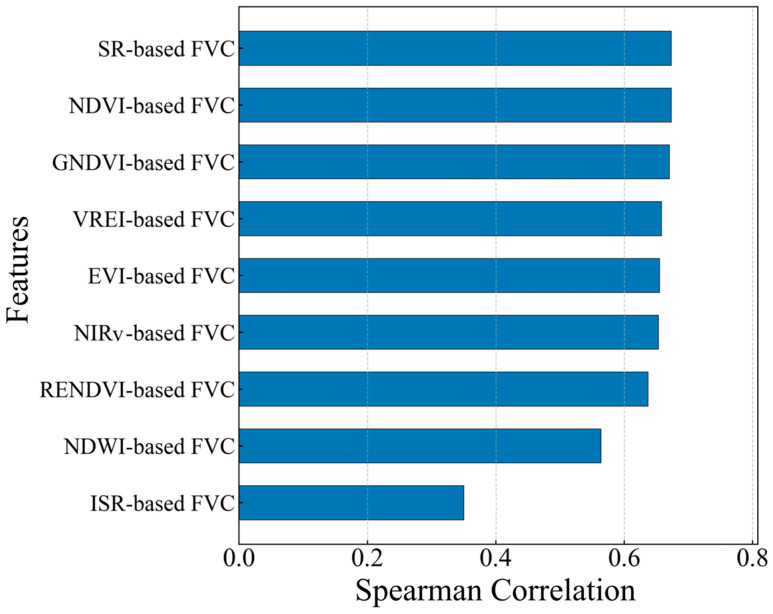
The Spearman correlation ranking of features.

When using machine learning for parameter inversion, excessive variables can increase model complexity and reduce computational efficiency. In this study, feature selection was performed by calculating the Spearman correlation between VI-based FVC and UAV-based FVC. The correlations among the vegetation coverages estimate a range from 0.35 to 0.67, as shown in Figure 6. The features with a Spearman correlation greater than 0.6 were selected and used as inputs to the machine learning models for vegetation coverage inversion. As a result, seven variables, including SR-based FVC, NDVI-based FVC, GNDVI-based FVC, VREI-based FVC, EVI-based FVC, NIRv-based FVC, and RENDVI-based FVC, were identified.

#### 3.2.2. Model Parameter Optimization

Model parameter optimization is critical for parameter inversion with machine learning models. This study employed Bayesian optimization to fine-tune the key parameters of RF, XGBoost, LightGBM, and DNN. The selected parameters, their predefined ranges, and their optimal values are shown in Table 2.

#### 3.2.3. Comparison of FVC Estimation Accuracy

**Figure 7:**
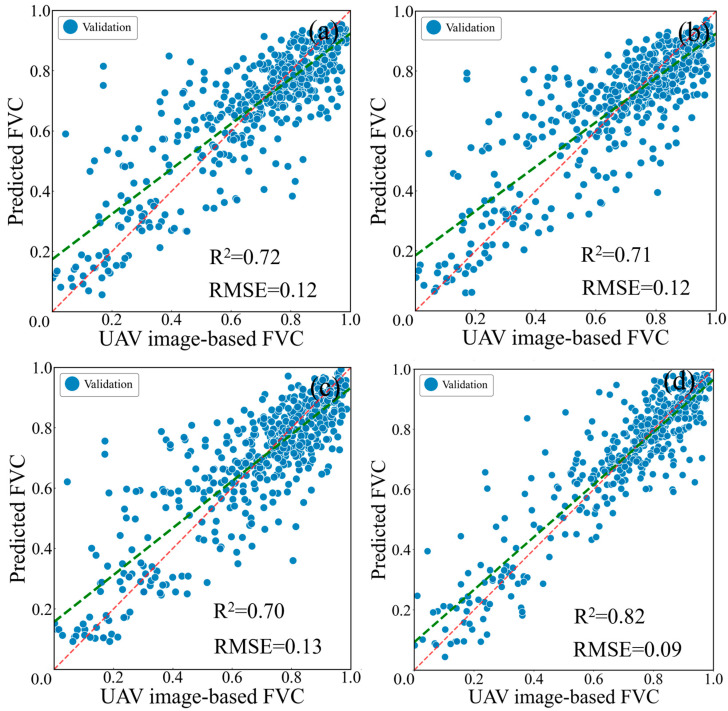
Comparison between UAV image-based FVC and predicted FVC using the four models. (**a**) RF; (**b**) XGBoost; (**c**) LightGBM; (**d**) DNN. The red dashed line and the green dashed line represent the 1:1 line and the regression line, respectively.

Based on the selected feature variables and optimized model parameters, FVC inversion was conducted using the RF, XGBoost, LightGBM, and DNN models. Ten-fold cross-validation was employed to evaluate model performance. The scatter plots of the UAV image-derived FVC versus the predicted FVC, along with the coefficients of determination and root mean square errors, are shown in Figure 7.

Figure 7 illustrates the FVC inversion performance of the four models. Overall, the machine learning model-based FVC estimates outperformed the pixel dichotomy model-based FVC estimates. For the validation dataset, all four machine learning models achieved coefficients of determination greater than 0.70 for FVC inversion, with the DNN model performing the best and reaching an R^2^ of 0.82. Regarding root mean square error, all four models achieved RMSE values no greater than 0.13, with the DNN model yielding the lowest RMSE of 0.09. Furthermore, the regression line of the FVC estimates derived from the machine learning models aligns more closely with the 1:1 line compared to that of the pixel dichotomy model, indicating that the machine learning approach avoids significant systematic overestimation in sparsely vegetated areas and underestimation in densely vegetated areas. Notably, the data points from the DNN model were more densely and uniformly distributed around the 1:1 line, reflecting its superior performance in FVC inversion.

## 4. Discussion

The Qinghai–Tibet Plateau alpine meadow vegetation coverage shows significant spatial heterogeneity and fragmented distribution characteristics. Conventional satellite image-based pixel dichotomy models struggle to capture this complex pattern accurately. Traditional remote sensing monitoring methods are limited by resolution and simplified model assumptions, often failing to reflect fine-scale changes in the alpine meadow vegetation. This study proposes an innovative method integrating UAV and Sentinel-2 data, obtaining vegetation coverage ground truth through ultra-high-resolution UAV imagery and combining machine learning algorithms to process the multi-source remote sensing data, which effectively improves the accuracy of alpine meadow vegetation coverage retrieval. The following section discusses the results of the study along with the advantages and limitations of the approach.

### 4.1. Pixel Dichotomy-Based FVC Estimation Using Different Vegetation Indices

This study evaluated the performance of nine vegetation indices in alpine meadow vegetation coverage retrieval. The results indicated that, in addition to the traditional NDVI-based pixel dichotomy method, other vegetation indices constructed using the near-infrared, red-edge, red, green, and shortwave infrared bands can also estimate vegetation coverage. For most of the vegetation indices (e.g., GNDVI, RENDVI, NIRv, EVI, SR, VREI), the coefficient of determination between the estimated FVC and the UAV-derived FVC reached approximately 0.65. Notably, the GNDVI-based FVC estimation achieved an R^2^ of 0.79, which was higher than that of the NDVI-based estimation at 0.67. To our knowledge, similar situations arose in previous studies. In the estimation of an aboveground biomass in the dryland forests of southern Africa, GNDVI demonstrated a higher coefficient of determination with the aboveground biomass than NDVI [41]. GNDVI also performed better in the classification of broadleaf and coniferous forests [42] and in the estimation of the leaf area index (LAI) [43]. The main reason for the performance difference between GNDVI and NDVI may be that NDVI is more sensitive to areas with low chlorophyll concentrations, while GNDVI is more responsive to areas with high chlorophyll concentrations [25]. Since vegetation coverage in the study area is mainly concentrated above 0.6, GNDVI yielded the best performance in estimating vegetation coverage.

Additionally, the study found that the performance of the FVC estimation using the different vegetation indices varied across areas with different levels of vegetation coverage. In low vegetation coverage areas (FVC < 0.4), the FVC estimates based on NIRv and SR demonstrated clear advantages, achieving an RMSE of 0.10. The NIRv index, in particular, effectively reduced soil background interference on vegetation signals through the product of near-infrared reflectance and NDVI, enhancing its vegetation detection capability under low coverage conditions [44,45]. In high vegetation coverage areas (FVC > 0.7), the FVC estimates based on GNDVI and RENDVI performed better, with RMSE values of 0.08 and 0.09, respectively. These two indices utilize the reflectance differences between the green (or red-edge) and near-infrared bands to more accurately quantify chlorophyll content and photosynthetic activity in densely vegetated areas [43]. Yan et al., using radiative transfer simulations to evaluate the vegetation coverage estimation based on the different vegetation indices, have similarly found that varying vegetation growth conditions can affect the estimation performance of the vegetation indices [10]. Therefore, integrating the estimates from multiple vegetation indices for vegetation coverage inversion tends to improve the estimation accuracy.

### 4.2. Feature Contribution Analysis of FVC Estimation Based on Machine Learning

**Figure 8:**
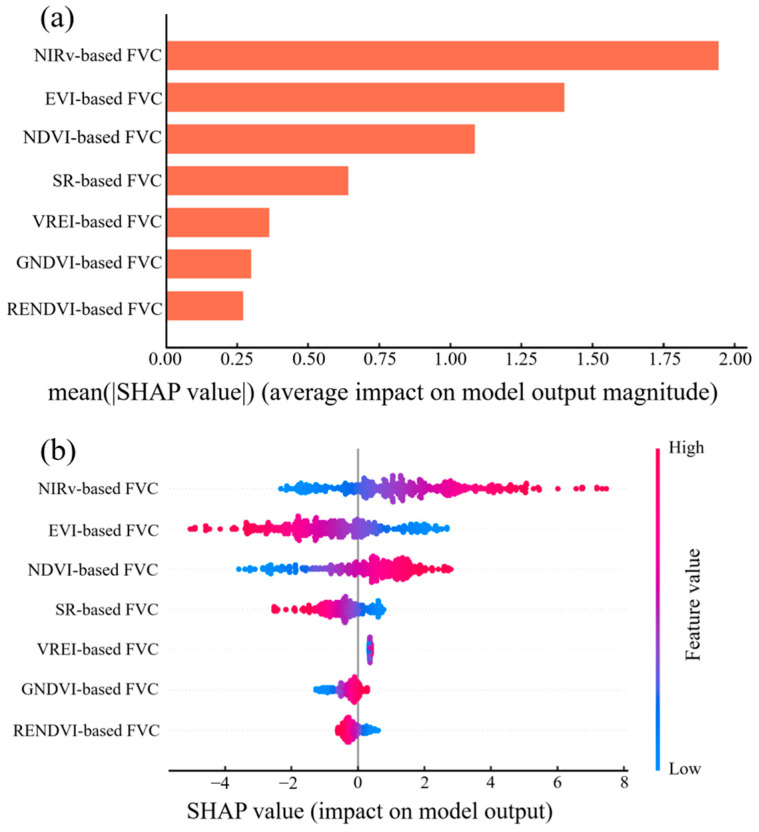
(**a**) Importance plot and (**b**) Summary plot based on the SHAP method.

This study evaluated four machine learning algorithms (RF, XGBoost, LightGBM, and DNN) for alpine meadow FVC estimations. The results indicated that all the machine learning models achieved higher FVC estimation accuracies than the pixel dichotomy model. The DNN model performed the best, with a coefficient of determination of 0.82 and a root mean square error of 0.09 for its estimation results. To explore the black box inversion process of the DNN model, the SHAP method was used to interpret each feature’s contribution to the DNN model’s estimation results. The Importance plot and Summary plot based on the SHAP method are shown in Figure 8.

Figure 8a presents the importance ranking of the various VI-based FVC indicators to the model output, calculated using the SHAP method. The horizontal axis represents the mean absolute SHAP value of each feature, indicating the average magnitude of its impact on the model predictions. As shown in the figure, NIRv-based FVC contributed the most to the inversion results in the DNN model, demonstrating the strongest explanatory power. EVI-based FVC, NDVI-based FVC, and SR-based FVC also played relatively essential roles in the model output. In contrast, the contributions of VREI-based FVC, GNDVI-based FVC, and RENDVI-based FVC were comparatively minor.

Figure 8b illustrates the specific direction and magnitude of the contribution of different FVC indicators to the model output. The row in the figure represents the input feature, and the horizontal axis shows the SHAP value for that feature in each sample, indicating its impact on the model output. A positive SHAP value means the feature increases the prediction, while a negative value indicates a decreasing effect. Each dot represents the SHAP value of a single sample, with the color indicating the actual value of the feature. As shown in the figure, NIRv-based FVC had the greatest contribution to the model output and exhibited a predominantly positive impact from high-value samples, indicating that higher NIRv-based FVC values tend to increase the model’s predictions. The EVI-based FVC, NDVI-based FVC and SR-based FVC showed a more bidirectional influence, with both high and low values distributed across the positive and negative SHAP value ranges. The SHAP values of the remaining features were mainly concentrated around zero, suggesting that their influence on the model output was relatively minor.

### 4.3. Advantages and Limitations

The joint retrieval method using UAV and satellite data proposed in this study offers several advantages for FVC estimation. First, it effectively addressed the difficulty of implementing traditional ground measurements in alpine meadow regions using centimeter-level UAV imagery, significantly reducing data collection costs and difficulties [46]. Second, this method integrated multiple vegetation index information through machine learning algorithms, fully utilizing the complementary advantages of different spectral features and overcoming the applicability limitations of single vegetation indices under different vegetation coverage conditions. Finally, the method established an effective scale conversion bridge from high-resolution UAV data to medium-resolution satellite data, providing a feasible approach for large-scale, high-precision vegetation coverage mapping.

Despite the strengths of the approach, there are also some limitations. First, in this study, the pure vegetation and pure bare soil pixels were identified based on the cumulative distribution thresholds of the vegetation indices when estimating vegetation coverage using the pixel dichotomy model. However, due to the diversity of the vegetation types and soil conditions, the spectral characteristics of the pure vegetation and bare soil pixels are complex [18]. Determining the vegetation and soil endmembers using empirical thresholds may introduce errors in the vegetation coverage estimations. Previous studies have indicated that the purity of the soil endmember plays a critical role in low vegetation coverage areas. In contrast, the purity of the vegetation endmember is more critical in high vegetation coverage areas. Moreover, when estimating vegetation coverage using the different vegetation indices, the impact of endmember purity on estimation accuracy varies [10]. In future work, we will determine the vegetation and bare soil endmembers using UAV imagery to improve the accuracy of the vegetation coverage estimations. Second, this study did not account for the influence of the solar elevation angle on the FVC estimations based on the pixel dichotomy model. As the solar elevation angle increases, a pixel may consist of four components: sunlit vegetation, sunlit soil, shaded vegetation, and shaded soil [8]. This violates the assumption of the pixel dichotomy model that a pixel contains only two components. Further, UAV-based multi-angle observations and radiative transfer simulations will be utilized to quantify the impact of the solar elevation angle and viewing angle on FVC estimation.

## 5. Conclusions

This study proposed a method for vegetation coverage retrieval by integrating Sentinel-2 and UAV data, effectively addressing the insufficient accuracy of traditional pixel dichotomy retrieval methods in the Qinghai–Tibet Plateau alpine meadow regions. The study demonstrated that by combining vegetation coverage extracted from centimeter-level UAV imagery with estimates derived from the pixel dichotomy model using Sentinel-2 imagery and multiple vegetation indices, the DNN model can achieve accurate vegetation coverage inversion in the alpine meadow regions. Notably, when applying the pixel dichotomy model for FVC estimation, the performances of the different vegetation indices varied depending on the level of vegetation coverage. The GNDVI performed better in areas with high vegetation coverage, while the NIRv and SR were more suitable for low-coverage areas. The advantage of the proposed method lies in achieving a reliable vegetation coverage inversion accuracy with minimal field survey effort, which is particularly valuable under the harsh environmental conditions of the Qinghai–Tibet Plateau. Future studies will focus on the influence of the endmember selection and solar elevation angle on the accuracy of FVC estimation.

## Figures and Tables

**Table 1:** The calculation formula and corresponding references for the vegetation indices.

Vegetation Indices	Calculation Formula	Reference
RENDVI	$\frac{NIR-RE}{NIR+RE}$	Gitelson and Merzlyak (1994) [23]
NDVI	$\frac{NIR-R}{NIR+R}$	Rouse et al. (1973) [24]
GNDVI	$\frac{NIR-G}{NIR+G}$	Gitelson et al. (1996) [25]
EVI	$2.5\;\frac{NIR-R}{NIR+6\times R-7.5\times B+1}$	Huete et al. (2002) [26]
ISR	$\frac{NIR}{SWIR}$	Fernandes et al. (2003) [27]
NIRv	$NIR\times \frac{NIR-R}{NIR+R}$	Tang L et al. (2021) [28]
SR	$\frac{NIR}{R}$	Birth and McVey (1968) [29]
VREI	$\frac{NIR}{RE}$	Vogelmann et al. (1993) [30]
NDWI	$\frac{GREEN-NIR}{GREEN+NIR}$	Gao (1996) [31]

**Table 2:** Model hyperparameters, value ranges, and optimal values in this study.

Model	Parameters	Range	Optimal Value
RF	n_estimators	[50, 1000]	959
	max_depth	[5, 30]	22
	min_samples_split	[2, 20]	6
XGBoost	learning_rate	[0.01, 0.3]	0.0108
	n_estimators	[50, 1000]	717
	max_depth	[3, 12]	5
LightGBM	learning_rate	[0.01, 0.3]	0.0204
	n_estimators	[50, 1000]	612
	max_depth	[3, 12]	12
DNN	learning_rate	[0.0001, 0.01]	0.0076
	layerDim	[2, 6]	6
	nodeDim	[32, 256]	256

## Data Availability

The data presented in this study are available on request from the corresponding author.
